# Exploring the Efficacy of Nonlinear Filters in CMOS for 2-D Signal Processing for Image Quality Enhancement

**DOI:** 10.3390/s24134213

**Published:** 2024-06-28

**Authors:** Hector Bandala-Hernandez, Alejandro Bautista-Castillo, José Miguel Rocha-Pérez, Victor Hugo Carbajal Gómez, Alejandro Díaz-Sánchez

**Affiliations:** 1 National Institute for Astrophysics, Optics and Electrónics, Luis Enrique Erro #1, Sta María Tonanzintla, Puebla 72840, Mexico; hector.hernandez@inaoe.edu.mx (H.B.-H.); jmiguel@inaoep.mx (J.M.R.-P.); victor.carbajalg@inaoep.mx (V.H.C.G.); adiazsan@inaoep.mx (A.D.-S.); 2 Benemérita Universidad Autónoma de Puebla, Facultad de Ciencias de la Electronica, Ciudad Universitaria, Mexico City 72000, Mexico

**Keywords:** CMOS filters, normalized squared error, median-median

## Abstract

This study rigorously investigates the effectiveness of nonlinear filters in CMOS for 2-D signal processing to enhance image quality. We comprehensively compare traditional linear filters’ performance, which operate on the principle of linearity, with nonlinear filters, such as the median-median (Med-Med) approach, designed to handle nonlinear data. To ensure the validity of our findings, we use widely accepted metrics like normalized squared error (NSE), peak signal-to-noise ratio (PSNR), and structural similarity index (SSIM) to quantify the differences. Our simulations and experiments, conducted under controlled conditions, demonstrate that nonlinear filters in CMOS outperform linear filters in removing impulse noise and enhancing images. We also address the challenges of implementing these algorithms at the hardware level, focusing on power consumption and chip area optimization. Additionally, we propose a new architecture for the Med-Med filter and validate its functionality through experiments using a 9-pixel image sensor array. Our findings highlight the potential of nonlinear filters in CMOS for real-time image quality enhancement and their applicability in various real-world imaging applications. This research contributes to visual technology by combining theoretical insights with practical implementations, paving the way for more efficient and adaptable imaging systems.

## 1. Introduction

The convergence of various technologies, such as 2-D signal processing, image filters, CMOS processors, and image quality assessment, has sparked a vibrant discourse in visual technology research [1,2]. This intersection is a meeting point of technologies and a battleground of challenges and opportunities, from fundamental theoretical foundations to practical applications. Two-dimensional signal processing, for instance, equips us with valuable tools for eliminating unwanted components [3]. Within 2-D arrays, the strategic application of linear and nonlinear filters in the spatial domain becomes a dynamic solution to filter out high-frequency undesired elements [1,4,5]. However, the conflict presented by linear filters, which are applicable in specific situations but result in blurring and information loss, prompts the investigation of nonlinear alternatives. The theoretical foundations of two-dimensional CMOS processors, conceptualizing them as processing elements (PEs) intricately woven into kernels within 2-D sensor arrays, is another area of intense research. The primary focus is addressing nonlinear non-uniformities among PEs arising from systematic and random mismatches [6,7]. This scenario explores various operations, customizing each to address issues arising from the statistical distribution of non-uniformities in each PE’s response. In the expansive realm of visual technology, filters play a pivotal role in shaping and enhancing digital images. From subtle smoothing adjustments to advanced edge-detection techniques, these tools are the backbone of image processing. Each filter serves a unique function, including smoothing, eliminating imperfections, edge detection, and highlighting critical structures.

Noise introduces challenges within digital imagery that demand nuanced, thorough understanding [8]. Fixed pattern noise (FPN), or spatial non-uniformity, refers to a consistent and predictable deviation from expected pixel values [9]. It often emerges because of imperfections or non-uniformities in imaging sensors. As image quality becomes paramount, introducing figures of merit for image quality offers a systematic approach to evaluating fidelity, clarity, and perceptual accuracy [10,11]. Metrics like the structural similarity index (SSI), peak signal-to-noise ratio (PSNR), normalized squared error (NSE), mean squared error (MSE), perceptual image quality assessment (PIQA), and structural similarity map (SSIM map) serve as benchmarks for assessing various facets of image quality [12]. This exploration seeks to comprehend linear and nonlinear filters, the role of 2-D-CMOS pixels, and the importance of image quality assessment metrics [13]. By comprehensively understanding and critically evaluating these elements, we aim to empower diverse fields with tools for consistently delivering and appraising superior visual content.

This paper is structured as follows. Section 2 analyzes the balance between quality improvement and energy consumption for different filter implementations. Section 3 describes the design of a weighted median filter (WMF) circuit for these multidimensional filters. Section 4 compares the performance of the WMF circuit with previous median filters. Section 5 explains the implementation of the Med-Med filter using a 9-pixel image sensor array. Section 6, presents the characterization setup. This is followed by Section 7, which presents experimental results. Finally, Section 8 concludes.

## 2. High-Level Simulation Applying Multilevel 2-D Filters

Three crucial metrics for assessing 2-D data quality, namely NSE, PSNR, and SSIM [7,11], are employed. Nonlinear contamination is introduced to the sample to validate the hypothesis, which is then processed using a mean filter, median filter, Med-Med, and Mean-Med techniques. These filters are not randomly chosen but specifically selected to correspond to the multilevel approximations explored in this study, underscoring their significance in our research.

**Figure 1:**
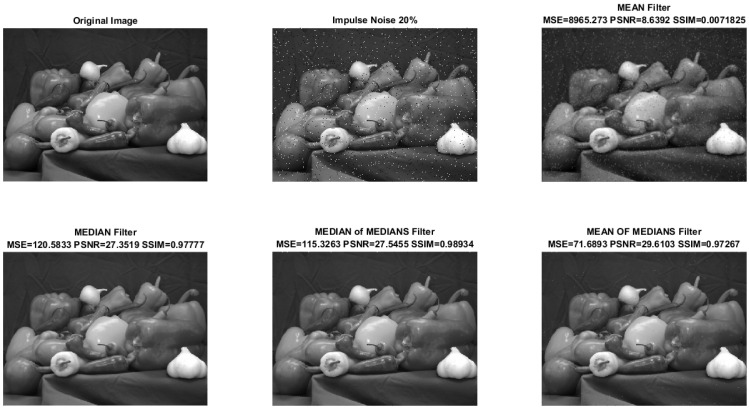
Performance of different 2-D filters on a grayscale image.

Simulations of the filters above were conducted on 509×383 pixels and 256 grayscale images, with the results depicted in Figure 1.

The original image is deliberately contaminated with 20% impulse noise, a level carefully chosen to assess performance under various working conditions. This deliberate choice of noise level ensures that our research covers a wide range of scenarios. Figure 1 illustrates the outcomes of mean, median, median of median, and mean of median filters, respectively.

Table 1 compares specific performance metrics between processed images and their unaltered counterparts, focusing on NSE, PSNR, and SSIM. As depicted in Table 1, SSIM ideally reaches 1; however, the mean filter exhibits a notably low value, attributed to the blurring effect observed when applied to a 2-D array. Furthermore, human perception of image quality in terms of SSIM is notable; notably, the image produced by the mean filter displays a low resemblance to the original image, which is evident in its low SSIM. Conversely, employing the Med-Med filter yields the highest SSIM.

**Figure 2:**
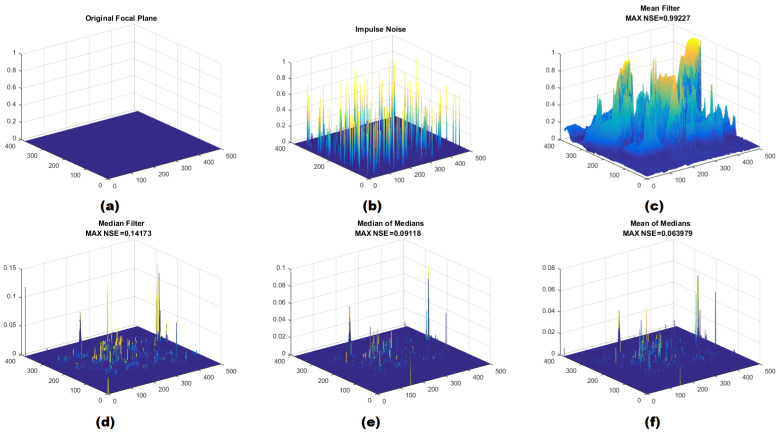
(**a**) Original image with focal plane, (**b**) Image with 20% impulse noise, (**c**) Filtered image using mean filter, (**d**) Filtered image using median filter, (**e**) Filtered image using median of medians filter, (**f**) Filtered image using mean of medians filter.

Moreover, normalized squared error (NSE) could significantly affect the perception of differences between the original and processed images. NSE necessitates the determination of the maximum pixel value. In our simulation, the variable $MAX_{i,j}$ is initialized to 256, representing the total number of grayscale levels within the image. The process depicted in Figure 2 illustrates the extraction of NSE, mirroring the procedure outlined in Figure 1.

Figure 2a demonstrates the results obtained by applying an ideal filter, resulting in an output image identical to the original, with a normalized squared error NSE=0. Following this, Figure 2b showcases a corrupted image with 20% impulse noise, while Figure 2c–f illustrate the NSE values of the applied filters. Noticeably, the NSE of the Med-Med and Mean-Med filters is significantly lower than that of both the mean and median Filters. In certain instances, the scaling in Figure 2 is adjusted to ensure perceptibility to the human eye.

**Figure 3:**
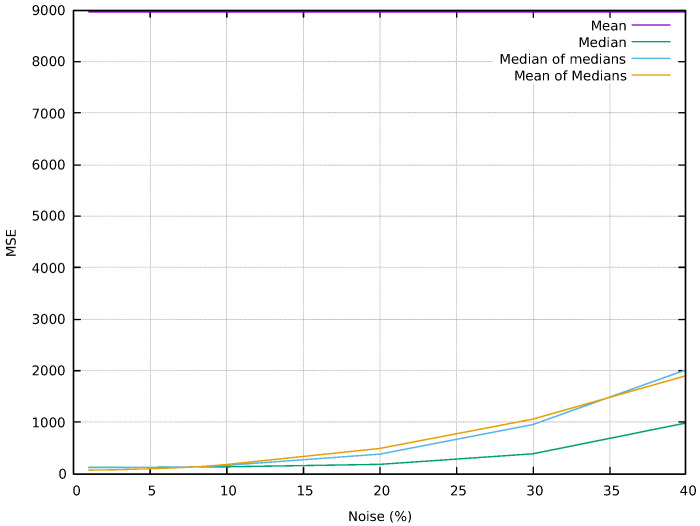
MSE comparing the filtered image to the noise.

Figure 3 shows the MSE behavior with four filters applied to an image contaminated with impulsive noise, showing that the mean filter is ineffective at any noise level, maintaining a consistently high MSE of around 8965. In contrast, the median and Med-Med filters exhibit better performance at low-to-moderate noise levels. However, their effectiveness diminishes as the noise increases, with the MSE rising from 120.1 to 983.1 and 114.1 to 2013.6, respectively. The Mean-Med filter performs best at low noise levels, with an MSE starting at 67.7 and increasing to 1900.7 at very high noise levels. Median-based filters are more effective at handling impulsive noise, particularly at lower levels, but their efficacy decreases with higher noise percentages.

### Power Constraints

**Figure 4:**
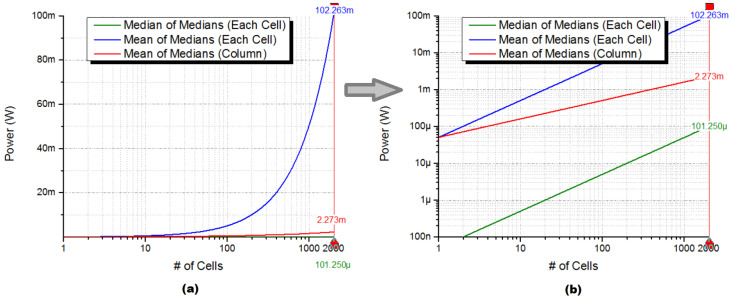
Power consumption of an IC implementation using Mean-Med and Med-Med algorithms: (**a**) logarithmic X-axis, (**b**) both logarithmic X- and Y-axes for improved visualization.

Considerations must be taken into account when implementing a low-power integrated circuit (IC), especially regarding power consumption. Figure 4 illustrates a comparison of the estimated power consumption between Med-Med and Mean-Med filters. In previous research [14], a low-power median filter was assessed and serves as a reference benchmark. The comparison in Figure 4 is based on the number of cells within a 2-D array. It is assumed that each median filter within a multilevel filter setup consumes 90 nW of static power per cell. The maximum power consumption depicted in Figure 4 is computed for an array consisting of 32×32 processing cells.

The observed compromise between enhancing quality and conserving power suggests that while the Mean-Med scheme provides better quality enhancement than the Med-Med algorithm, implementing it on each cell increases power consumption as the number of cells grows. Consequently, when dealing with extensive 2-D arrays, opting for column-wise filtering over deploying a processing element on each cell is preferable to alleviate power usage. This method capitalizes on the inherent parallelism in column-wise operations, thereby reducing the overall power requirements.

On the other hand, the Med-Med algorithm emerges as a more fitting solution for achieving superior quality enhancement without sacrificing low-power characteristics. Its inherently localized nature enables efficient processing without substantially escalating power demands, rendering it an appealing choice for applications where power efficiency is crucial.

## 3. Hardware Implementations of the Algorithms

Initially, attention is directed towards the fundamental design of the Mean-Med and Med-Med filters, specifically the circuit responsible for extracting the median from a set of values [15,16,17]. This median extractor circuit is versatile and can operate as a weighted median extractor, enhancing its applicability across various scenarios.

### Median Extractor and Weighted Median Filter

The weighted median filter (WMF) is defined by a function of the estimator β, which minimizes the weighted $L_{1}$ norm, as depicted in Equation (1) [18,19,20].

(1) $$\Sigma_{i=1}^{N}w_{i}\left|x_{i}-\beta \right|\to min\;\;\;\;i,w_{i}\in N$$

In this context, *N* represents the number of inputs, $x_{i}$ represents the *i*-th entry of a continuous-valued input vector, and $w_{i}$ denotes the weight coefficient vector, where min represents the statistical minimum. Each element in the weight vector $W=w_{1},\ldots w_{N}$ must be a real, non-negative integer, and the sum of the components must be an odd number.

**Figure 5:**
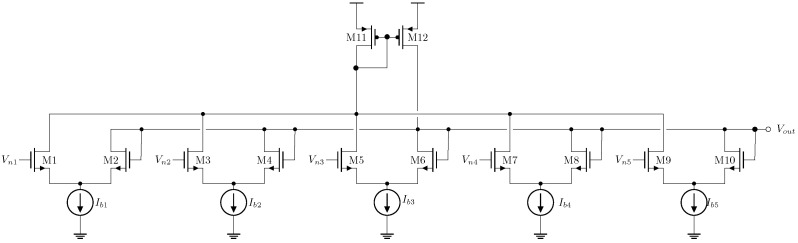
The 5-input median extractor circuit.

The depicted median circuit extractor in Figure 5 employs a transconductance comparator per datum. The proposed weighted median filter (WMF) topology can be realized by utilizing the tail current to establish different saturation levels and assign weights to each input. Each $I_{bn}$ represents a non-negative multiple of a base current $I_{x}=10$ nA. Thus, if $I_{b1}$ to $I_{b5}$ are 10 nA, the circuit operates as a standard median extractor. The sub-window schematic circuit of this non-weighted median extraction is:

For this circuit, operational transconductance amplifiers (OTAs) with input data exceeding $v_{out}$ will saturate positively, resulting in positive saturation currents $+I_{out_{i}}$ flowing into node $v_{out}$. Conversely, for OTAs with input data below $V_{out}$, the output currents will yield negative output saturation currents $-I_{out_{j}}$ from node $V_{out}$. Maintaining equilibrium, the number of weighted data below the median must be consistent, as illustrated in Equation (2).

(2) $$\Sigma_{i}I_{out_{i}}-\Sigma_{j}I_{out_{j}}\approx 0$$

For all tail currents except that of the comparator whose input is the median of the set of inputs, the output voltage, $V_{out}$, of the corresponding OTA closely approximates $v_{in}$. In any weighted median filter where the sum of the weights is an odd number, the output voltage tracks the corresponding input in the dataset. All comparators in the proposed filter operate in the subthreshold region and utilize $I_{bn}$ as the tail current when a non-weighted median is desired, resulting in a power consumption of 90 nW for the entire WMF cell. When a weighted median is required, the power consumption is calculated using Equation (3) [21,22,23].

(3) $$P_{WMF}=18nW*\Sigma_{i}^{n}w_{i}$$

Table 2 displays the assigned transistor sizes of the weighted median circuit in Figure 5, the bias current, and the supply voltage of the median circuit extractor.

**Figure 6:**
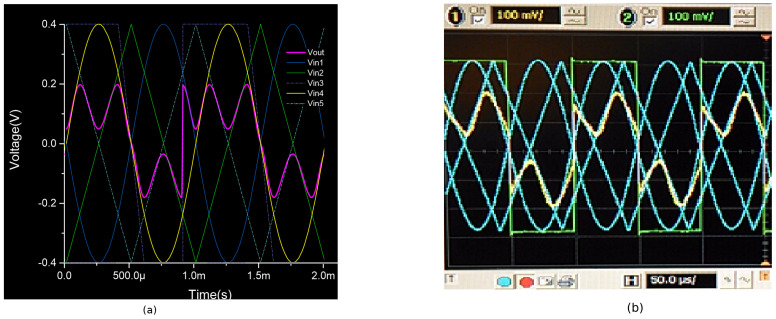
Transient response of the 5-input median filter applying the mask W={1,1,1,1,1}: (**a**) simulation results, (**b**) experimental results, 400 mVpp signals at 10 KHz.

The weight vector of the WMF can be represented as:

(4) $$I_{bn}=I_{x}w_{n}\;\;\;\;n=1,2\ldots 5$$

where $w_{n}\;\;\in \;\;N$ and is the weight used for the *n*-th datum. Although the comparator transconductance could vary due to differences in the tail currents, the filter’s performance remains unaffected. A weighted median extraction example is depicted in Figure 6, where the mask $W_{b}=\left\{3,1,1,1,3\right\}$ is applied. Both median and weighted median extractors function as the core block for the implemented multilevel filters in this study. Subsequent sections detail the design of various multilevel filter implementations.

## 4. WMF Experimental and Simulation Results

The WMF circuit was fabricated using a 0.5 μm technology provided by ON Semiconductors. Table 3 details the five inputs utilized for characterizing the chip. Figure 6b illustrates the experimental response of the median circuit, employing the weight mask W={1,1,1,1,1}, with all comparators biased at a current of 10 nA. Discrepancies between the corners of the simulated output signal (pink line) and those of the experimental output (yellow line) are observed; the experimental results do not fully reach the corners corresponding to the ideal median due to a single comparator being assigned to each resulting median. However, in cases where the median aligns with such corners, the circuit needs to switch between the current and the subsequent comparator.

**Figure 7:**
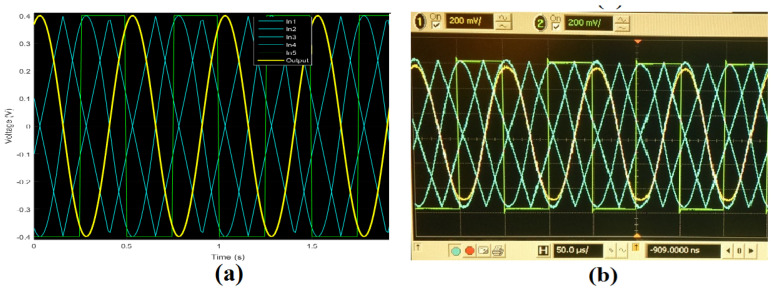
WMF response applying mask $W_{a}=\left\{3,1,1,1,1\right\}$: (**a**) MATLAB simulation and (**b**) experimental results, 400 mVpp signals at 10 KHz.

The five patterns of input window covers utilized during the weighting procedure are indicated as $\left\{w_{1},w_{2},w_{3},w_{4},w_{5}\right\}$, where $w_{n}$ symbolizes the weight coefficient for the *n*-th input. The cover pattern $W_{a}=\left\{3,1,1,1,1\right\}$ was utilized with the WM filter. This assigns a superior weight to input $V_{in1}$ when compared with the remaining inputs. Within this pattern, the digit 1 represents the standard value of 10 nA for the tail current of the respective comparator, while the digit 3 corresponds to a tail current of 30 nA for the weighted transconductance comparator. MATLAB simulation outcomes employing cover $W_{a}$ are illustrated in Figure 7a, where five waveforms (two sine waves, two triangular waves, and a square wave) are utilized for an ideal WMF; the yellow waveform portrays the weighted median outcome. Furthermore, Figure 7b demonstrates experimental outcomes of the identical cover application. In this evaluation, the yellow waveform represents the WMF outcome, where five 10 KHz/400 mVpp waveforms were employed for the simulation outcomes and 10 KHz/600 mVpp for the experimental outcomes, in alignment with the earlier scenario.

The current mode technique of the described circuit enable overcoming certain constraints of analog median filters reported previously [14,18,20]. This comparison is presented in Table 4, which assesses latency, silicon area, power consumption, supply voltage, operational frequency, technology, input count, comparator gain, accuracy, type (analog or digital), and input common mode range (ICMR).

Based on Table 4, this study necessitates minimal silicon area and significantly reduces power consumption compared with other research efforts, rendering it appropriate for ultra-low power applications. The proposed analog WMF underwent validation through simulations utilizing the BSIM3 Level 49 model for a one-dimensional signal and two weight masks. Discrepancies between simulation and experimental outcomes arise from constraints on the output signal’s corners, influenced by the switching between saturated comparators operating concurrently. To mitigate such discrepancies, increasing the transconductance of the comparators may be necessary.

## 5. 9-Pixels Image Sensors Array for Median of Medians

**Figure 8:**
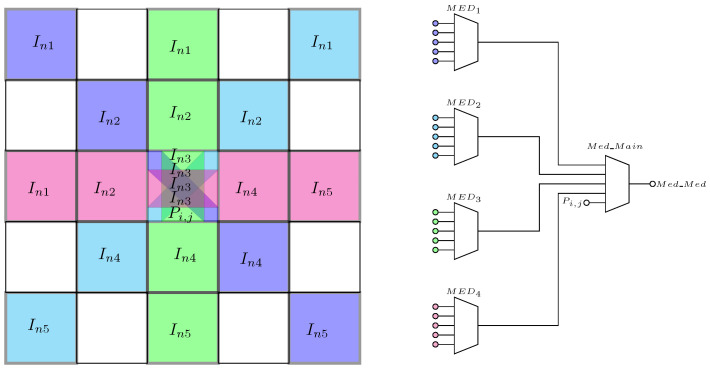
Scheme for the multilevel filter for median of medians extractor.

According to the high-level simulations performed in Figure 1 and Figure 2, the Med-Med filter demonstrates intermediate improvements in NSE, PSNR, and SSIM. Consequently, the hardware implementation of the Med-Med filter is shown in Figure 8 [24,25,26]. The filter comprises the 5-input median extractor circuit, as depicted in Figure 5. Therefore, the pixel array must be configured as a 5 × 5 grid and applied in the following manner. The first median filter processes pixels from the top-right corner to the bottom-left corner, the second median filter processes pixels from the top-left corner to the bottom-right corner, the third median filter processes the central column, and the fourth median filter processes the central row. The outputs of these four filters are connected to a fifth median filter, with the fifth input of the filter also processing the central pixel of the array. Consequently, this arrangement constitutes the Med-Med filter.

**Figure 9:**
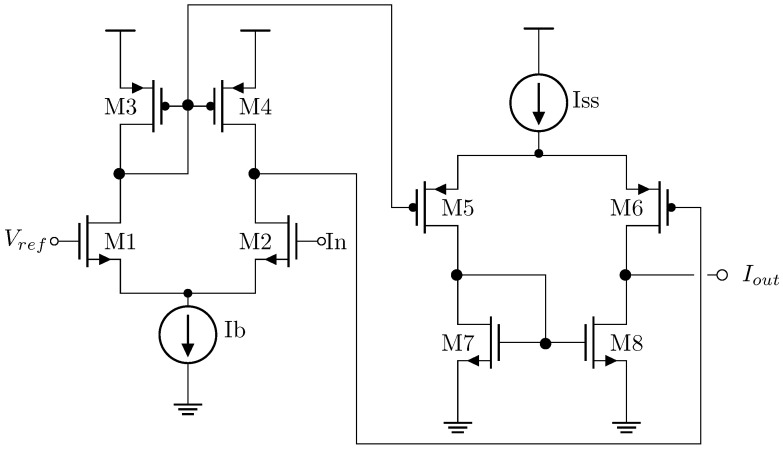
Schematic circuit of the analog comparator in the Mean-Med implementation for image sensors.

The VLSI implementation of this algorithm was fabricated using AMS 0.35 μm Opto technology within a multi-project wafer. Due to design area limitations, the 5 × 5 array could not be implemented, and a 3 × 3 array was utilized instead. To maintain the functionality of the 5-input median extractor circuit, the $I_{n1}$ inputs were connected to $V_{DD}$ and the $I_{n5}$ inputs were connected to $V_{SS}$. Each pixel consists of a 3 TAPS (3-transistor active pixel sensor). Since this array does not feature a correlated double sampling (CDS) system, a 4 TAPS (4-transistor active pixel sensor) was not implemented. The circuit employs a two-stage analog comparator, as illustrated in Figure 9 [27,28].

The low-frequency voltage gain of the comparator is show in Equation (5)

(5) $$\frac{V_{out}}{V_{in}}=gm_{1}gm_{5}\left(r_{o4}∥r_{o2}\right)\left(r_{o6}∥r_{o8}\right)$$

The initial phase comprises a differential pair employing NMOS transistors at its differential input and a PMOS transistor current mirror serving as an active load. The subsequent phase incorporates another differential pair utilizing PMOS transistors at its input and an NMOS transistor current mirror as an active load. The transistor type selection at each stage’s input aims to address the offset in the DC level voltage at the output. Equation (6) define the $r_{o,n}$ resistance.

(6) $$r_{o,n}=\frac{1}{\lambda I_{D}}$$

Subsequently, a tail current of 20 μA is suggested for the initial stage, while 10 μA is proposed for the second stage.

Table 5 shows the characteristics of the comparator.

## 6. Characterization Set-Up

**Figure 10:**
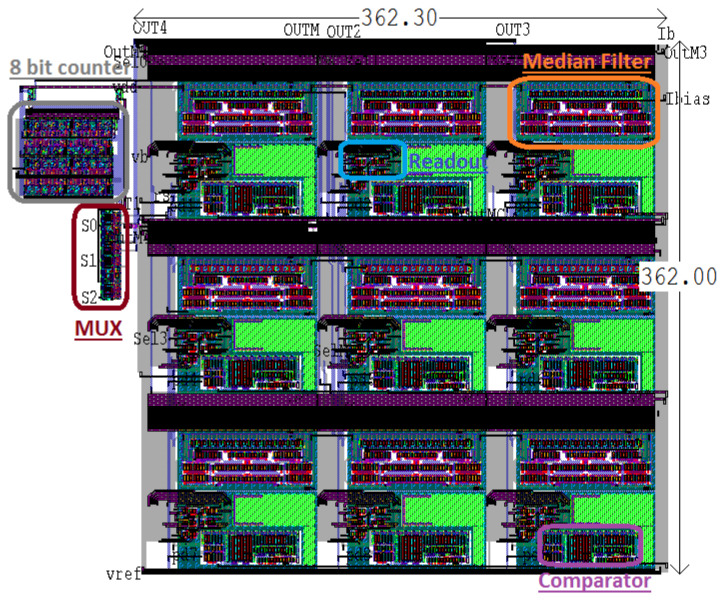
The 3 × 3 sensor array for the Med-Med filter accomplishment. CMOS Opto Technology.

**Figure 11:**
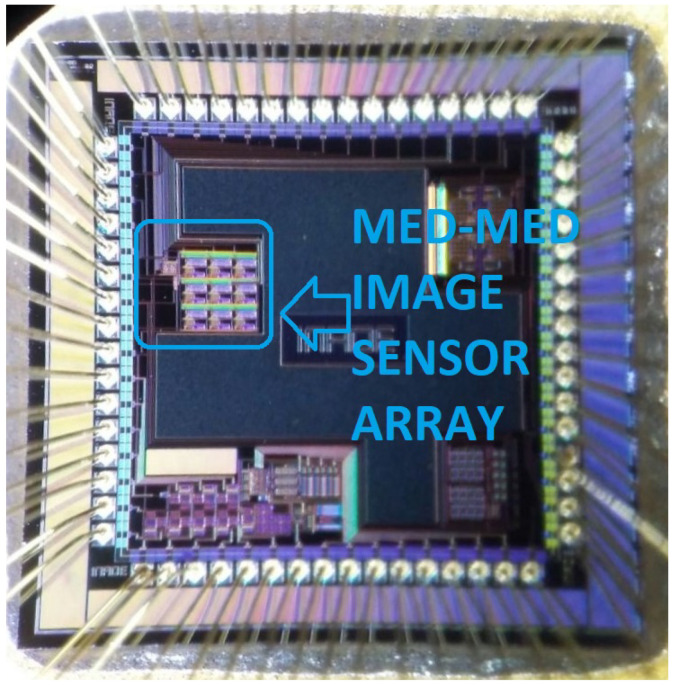
Photography of 3 × 3 sensor array for the Med-Med filter accomplished using AMI 0.35 μm CMOS Opto Technology.

Firstly, Figure 10 shows the layout of the 3×3 sensors array for the Med-Med filter accomplishment. Each section of the array is composed of a 5-input, subthreshold median filter used as sub-filter for the Med-Med filter, 3-transistors active pixel sensor, comparator, and a bias cell that supplies a current of 12.5 nA to each WMF. Figure 11 depicts a micro-photograph showcasing the 3×3 sensor array tailored for the Med-Med filter. The fabrication process leveraged AMI’s 0.35 μm CMOS Opto Technology, ensuring precise and efficient functionality.

**Figure 12:**
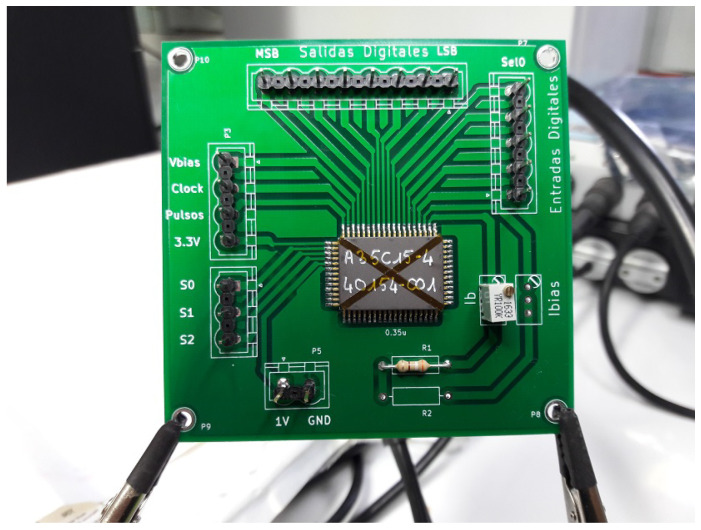
PCB for experimental measurements of Med-Med implementation.

**Figure 13:**
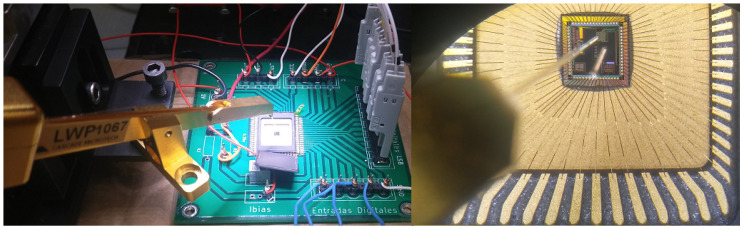
Alignment of the optical fiber to Med-Med implementation.

A meticulously designed and fabricated PCB (printed circuit board), as depicted in Figure 12, was utilized to complement this characterization of the Med-Med IC implementation. Concurrently, optic excitation parameters were configured to effectively apply mask patterns across the 3×3 array. For this purpose, a laser operating at 750 nm wavelength, coupled with an optic fiber, was employed to generate a spot size of 150 μm (as illustrated in Figure 13).

The experimental arrangement, as depicted in Figure 13, comprises diverse elements. The left side features a micro-manipulator containing the optic fiber, alongside the PCB, as illustrated in Figure 13. This PCB connects to a Keysight 16851A logic analyzer, enabling the capture of associated output digital words. Conversely, the right side of the image provides a microscopic view, demonstrating the accurate positioning of the fiber onto the chip.

## 7. Experimental Results

The pixels of the 9-pixel array were illuminated using an optical fiber termination. The fiber was aligned manually, with the moving capability along the X and Y directions across the array. Due to alignment imperfections, the fiber was positioned as centrally as possible over the desired pixel, although some of the incident light reached adjacent pixels. Consequently, the pixel values were measured manually, one by one, to obtain the median sub-filter values, their outputs, and, ultimately, the output of the main median filter centered on the image below.

### Results for $Msk_{2}$

**Figure 14:**
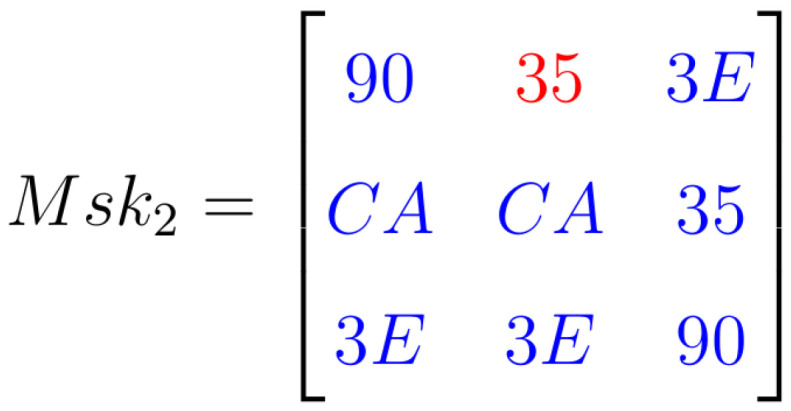
Mask 2 projected onto the 9-pixel array for Med-Med filter.

**Figure 15:**
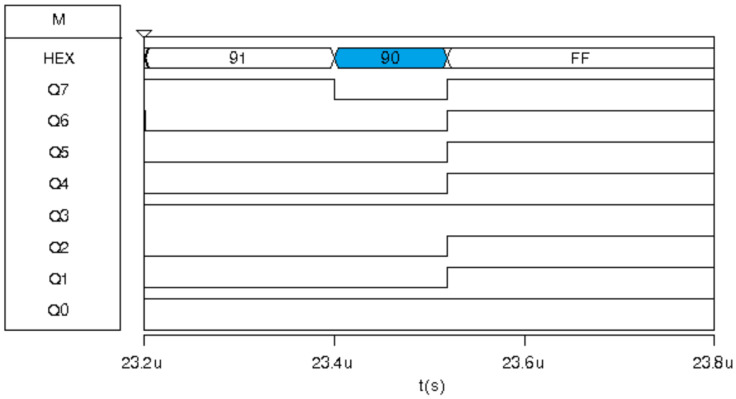
Digital response for the Med-Med image sensor prototype for the $Msk_{2}$ exposure corresponding to $MED_{F1}$.

Msk2 refers to the illumination pattern of the 3 × 3 pixel array centered at position (1,2), resulting in a HEX 35 value for this pixel, color red in Figure 14. Due to the lower intensity of illumination in the neighboring pixels, different values are obtained (Figure 14). In this instance, the output of the Med1 filter is HEX 90 (Figure 15).

$MED_{F1}$ sub-filter has inputs: {90,CA,90}; therefore, its output is ninety, as shown in Figure 15.

**Figure 16:**
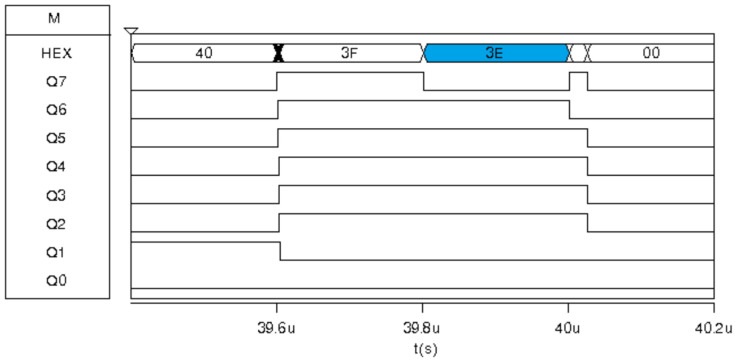
Digital response for the Med-Med image sensor prototype for the exposure corresponding to $Msk_{2}$: $MED_{F2}$ and $MED_{F3}$.

The $MED_{F2}$ and $MED_{F3}$ filters in $Msk_{2}$ have the same result as their respective sub-filter. $MED_{F2}$ has inputs {35,CA,3E}; therefore, its median is 3E. Similarly, inputs for $MED_{F3}$ are {3E,CA,3E}, resulting in the HEX 3E value. This result is shown in Figure 16 highlighted in blue.

**Figure 17:**
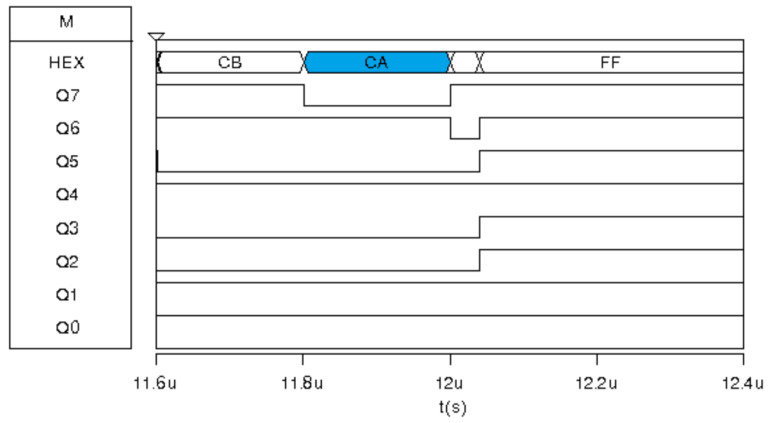
Digital response for the Med-Med image sensor prototype for the $Msk_{2}$ exposure corresponding to $MED_{F4}$.

Inputs for $MED_{F4}$ sub-filter applying $Msk_{2}$ are {CA,CA,35}; hence, the corresponding output is CA, as shown in Figure 17.

**Figure 18:**
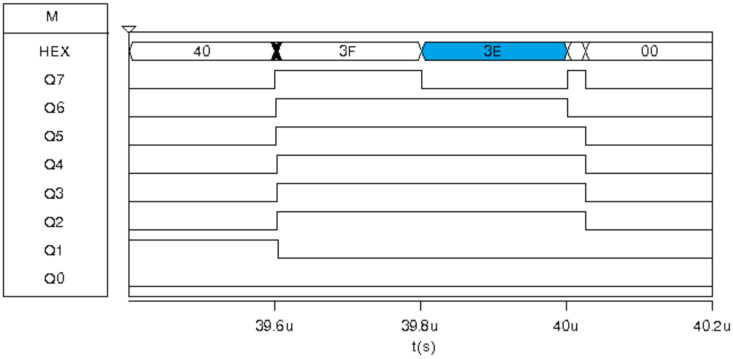
Digital response for the Med-Med image sensor prototype for the $Msk_{2}$ exposure corresponding to $MED_{Fmed}$.

Finally, inputs for the highest hierarchy median filter are {90,3E,3E,CA,3E}, resulting in a Med-Med of 3E, shown in Figure 18.

Having accessed each of the values, the functionality of the Med-Med filter was verified. This confirms that the hardware-level implementation is performing real-time filtering with filters consuming 90 nW.

Table 6 provides a detailed comparison between our proposed methodology and three alternative approaches within the field. While each method presents unique features, our solution, developed using AMS 0.35 μm technology, offers several significant advantages. Primarily, it showcases power efficiency, with power consumption as low as 450 nW during Med-Med operation when all five filters are active, and only 90 nW for a single median filter at 500 KHz with 1.8 Vpp, making it highly suitable for energy-constrained applications. Furthermore, our methodology has a dynamic range spanning from 0.1 V to 1.7 V, ensuring versatility in signal processing, coupled with an accuracy of 40 mV. Additionally, our design, featuring a WMF operating in the subthreshold regime, promises robust performance across varied scenarios. In contrast, the alternatives vary in their offerings; for instance, ref. [29] present a pixel array design with a single median filter, providing a different trade-off between power consumption and performance. Ref. [30] explore the use of comparators, while [17] implement a median filter, each with its strengths and limitations. However, our methodology sets itself apart by requiring no data sorting, simplifying the processing pipeline, and occupying a silicon area of 0.013 mm${\;\;}^{2}$, making it space-efficient and cost-effective. Notably, our implementation encompasses a holistic solution, incorporating a 9-pixel array with photosensors, readout components, comparators, median filters, and digital logic for reading, ensuring comprehensive signal processing in imaging applications.

## 8. Conclusions

This study comprehensively explores the intersection between 2-D signal processing and image quality assessment, focusing on the crucial role of linear and nonlinear filters alongside the design and implementation of two-dimensional CMOS processors. Through simulations, practical experiments, and theoretical analyses, we have showcased that these nonlinear filters, particularly the Med-Med approach, offer substantial improvements in NSE, PSNR, and SSIM compared with conventional linear filter-based methods.

Implementing these algorithms in integrated circuits poses additional challenges, including power management and chip area optimization, which are essential. While the Mean-Med approach may offer notable enhancements in image quality, its integration into low-power circuits could incur prohibitive energy consumption costs. Conversely, the Med-Med approach delivers significant image quality improvements without excessively compromising power efficiency.

Moreover, we introduced a novel Med-Med filter architecture realized in a 9-pixel image sensor array and confirmed its functionality through practical experiments. The obtained results affirm the efficacy and feasibility of this architecture in real-world applications and highlight its potential for integration into computer vision systems and imaging devices.

The conclusions drawn from our analysis show the positive impact of the Med-Med filter on image quality. With a notably low NSE of 115.2, the Med-Med filter significantly outperforms both mean and median filters regarding restoration accuracy, showing minimal discrepancy between the original and restored images, translating to superior visual fidelity.

Furthermore, the PSNR of 27.5 achieved with the Med-Med filter confirms its efficacy in preserving image quality, as higher values denote minimal information loss during restoration. Conversely, mean and median filters show inferior PSNR performance, suggesting a more substantial degradation of image quality.

The structural similarity index (SSIM) of 0.988 reached with the Med-Med filter further underscores its ability to preserve the structure and details of the original image, showing high similarity between the restored and reference pictures and confirming the effectiveness of the Med-Med filter in keeping crucial visual information.

In summary, the results highlight the superiority of the Med-Med filter over mean and median filters concerning restoration accuracy and preservation of image quality. Its ability to reduce NSE, increase PSNR, and keep high SSIM positions it as a highly effective choice for image restoration applications where visual quality is paramount. This study underscores the significance of interdisciplinary research in visual technology, emphasizing the necessity to address theoretical challenges and practical considerations for advancing more efficient, exact, and adaptable imaging systems with broad applications across various industries.

## Figures and Tables

**Table 1:** Mean squared error, peak signal-to-noise ratio, and structural similarity index are evaluated for a clean image compared with an image corrupted with 20% impulse noise.

Measure\Filter	Mean	Median	Med-Med	Mean-Med
MSE	8965.3	120.9	115.2	71.2
PSNR	8.6	27.3	27.5	29.6
SSIM	0.007	0.977	0.988	0.971

**Table 2:** **Dimensions of 5-input median extractor circuit.**

${\left(W/L\right)}_{M1\ldots M10}$	${\left(W/L\right)}_{M11-M12}$	$I_{x}$	$V_{\mathrm{DD}}$
40 μm/1.2 μm	60 μm/1.2 μm	10 nA	1.8 V

**Table 3:** Input signals of the median extractor circuit.

WFM Input	Signal (400 mVpp)	Frequency
$v_{in1}$	Sinusoidal	10 kHz
$v_{in2}$	Sinusoidal (90°)	10 kHz
$v_{in3}$	Triangular (45°)	10 kHz
$v_{in4}$	Triangular (45°)	10 kHz
$v_{in5}$	Square	10 kHz

**Table 4:** Median filters comparison.

	[18]	[16]	[19]	This Work
Latency	4.4 ns	34 ns	-	5 μs
Silicon Area	0.03 mm${\;\;}^{2}$	0.014 mm${\;\;}^{2}$	0.004 mm${\;\;}^{2}$	0.013 mm${\;\;}^{2}$
Power	13.5 μW	-	1.25 mW	90 nW
V-Supply	3 V	3.3 V		1.8 V
$Freq@C_{L}$	10 kHz @ 10 pF	10 MHz @ ?	-	500 kHz @ 10 pF
Technology	0.5 μm	0.35 μm		0.5 μm
# of Inputs	3	9	3	5
Comparator Gain	-	3800 V/V	-	100 V/V
Accuracy	-	10 mV	0.4 μA discriminability	40 mV
Type	Analog VLSI			
ICMR	−0.9 V to 1.5 V	0 V to 1.8 V	-	0.1 V to 1.7 V

**Table 5:** **Comparator characteristics.**

${\left(W/L\right)}_{1,2,7,8}$	${\left(W/L\right)}_{3,4}$	${\left(W/L\right)}_{5,6}$	$Power_{diss}$	$Sett_{time}$	$V_{supply}$
9 μ/0.9 μ	3 μ/0.9 μ	16 μ/0.9 μ	385.63 μW	9 ns	±1.65 V

**Table 6:** Array comparison.

	This Work’s Median Filter	[29] (Pixel Array, 1 Median Filter)	[30] (Comparator)	[17] (Median Filter)
Technology	AMS 0.35 μm	0.35 μm	AMS 0.35 μm	TSMC 0.18 μm
Comparator gain	100 V/V	3800 V/V	>1000 V/V	Not reported
Vsupply	1.8 V	3.3 V	3.3 V	2 V (vdd = vss = 1V)
Power	450 nW @Med-Med operation (5 filters active), 90 nW (single median filter) @500 KHz, 1.8 Vpp	1.25 mW	7–22 μW	Not reported
Dynamic range	0.1 V–1.7 V	0–1.8 V	Not reported	0–255 μA
Type of filter	Weighted analog median—Subthreshold	Analog median filter *	N/A	Analog median filter *
number of inputs	Med-Med mode = 21, Standalone = 5	9	N/A	9
Lab measurements	Yes	Yes	Simulations	Simulations
Data sorting required	No	Yes **	N/A	Yes
Accuracy	40 mV	10 mV	N/A	Not reported
Silicon area	0.013 mm${\;\;}^{2}$	0.014 mm${\;\;}^{2}$	Not reported	Not reported
Additional comments	9-pixel array, including photosensors, readout, comparator, median filters, and digital logic for reading	32 × 32 pixel array with external FPGA logic and single median filter for the whole array	Explores the use of analog comparators in CMOS imagers	A median circuit implementation

* MAX-MIN circuit required. ** Median operation is performed after readout via one median filter for whole array.

## Data Availability

The study did not report any data.
